# Virtual connection and real community: the qualitative experience of participating in a videoconferencing-based psychotherapy group for postpartum depression and anxiety

**DOI:** 10.1186/s12913-024-11753-y

**Published:** 2024-11-01

**Authors:** Neesha Hussain-Shamsy, Amika Shah, Lori Wasserman, Greer Slyfield Cook, Kaeli Macdonald, Keisha Greene, Geetha Mukerji, Simone N. Vigod, Juveria Zaheer, Emily Seto

**Affiliations:** 1 Institute of Health Policy, Management and Evaluation, University of Toronto, 155 College St 4th Floor, Toronto, ON M5T 3M6 Canada; 2 Women’s College Hospital, 76 Grenville St, Toronto, ON M5S 1B3 Canada; 3 Centre for Digital Therapeutics, University Health Network, R. Fraser Elliott Building, 4Th Floor, 190 Elizabeth Street, Toronto, ON M5G 2C4 Canada; 4 Women’s Institute of Health Systems Solutions and Virtual Care, Women’s College Hospital, 76 Grenville St, Toronto, ON M5S 1B3 Canada; 5 Department of Medicine, Temerty Faculty of Medicine, University of Toronto, 1 King’s College Cir, Toronto, ON M5S 1A8 Canada; 6 Department of Psychiatry, Temerty Faculty of Medicine, University of Toronto, 1 King’s College Cir, Toronto, ON M5S 1A8 Canada; 7 Centre for Addiction and Mental Health, 250 College St, Toronto, ON M5T 1R8 Canada

**Keywords:** Postpartum, Depression, Anxiety, Group therapy, Virtual care, Qualitative

## Abstract

**Background:**

Group psychotherapy, an effective treatment for common postpartum mental disorders (e.g. depression, anxiety), has increasingly been delivered virtually since the pandemic. This study aims to understand experiential aspects of participating in videoconferencing-based group psychotherapy in the postpartum period.

**Methods:**

Our urban academic ambulatory hospital has delivered group psychotherapy for women (cis and trans) and non-binary individuals of female sex with postpartum depressive and anxiety symptomatology via videoconferencing since 2020. One therapist-facilitator conducts weekly 60-min group therapy sessions with 5-6 participants for eight weeks. Group participants were invited to complete a semi-structured qualitative interview on their experience. Using an interpretive description approach, we conducted reflexive thematic analysis to code anonymized transcripts and construct themes. Facilitator interviews were used for triangulation and additional context.

**Results:**

Of 134 patients in video psychotherapy groups over 11 months, 14 completed an interview, as did all group facilitators (*n* = 3). Overall, participants felt the experience with videoconferencing group therapy was beneficial for their mental health. Three themes were constructed: (1) “Moving Towards a New Normal”: The group helped participants normalize feelings and experiences around transition to parenthood, and accessing health care virtually was now considered to be normal practice, although some wanted an element of choice. (2) “Virtual Connection, Real Community”: Connections were made virtually, yet participants felt a real sense of community. Facilitators played an important role fostering an environment in which participants could create lasting bonds. Participants noted challenges with feeling comfortable virtually and provided pragmatic and structural suggestions for enhancing the creation of community. (3) “Trade-offs to Virtual Engagement”: Participants made positive and negative trade-offs (e.g. no informal interactions, travel, isolation at home, childcare challenges) to maximize their experience and were able to be more authentic in their self-presentation to the group.

**Conclusions:**

People with postpartum depression and anxiety who participated in videoconferencing-based group psychotherapy appreciated the sense of community within their groups to normalize their experience transitioning to parenthood. Participants had to make trade-offs to access virtual groups, but felt the experience was worthwhile and helped improve their mental health. Findings will help inform continued delivery of virtual group mental health services.

**Supplementary Information:**

The online version contains supplementary material available at 10.1186/s12913-024-11753-y.

## Background

Common postpartum mental disorders such as depression and anxiety can be effectively treated using psychological interventions (i.e. formalized, therapeutic interventions) either individually or in a group setting [1–3]. Group therapy, where a small group of patients receive the same therapeutic intervention from a trained therapist or facilitator, offers a number of advantages compared to individual treatment at the system (e.g. cost effectiveness, resource efficiency, etc.) and individual (e.g. shared learning opportunities, modelling others’ behaviours, normalizing experiences, improving social support) levels [4–8]. The group environment and the experience that is co-created by the group members and facilitator(s) play important roles in influencing treatment outcome, ensuring the provision of evidence-based care, and safeguarding against negative experiences [9–12].

The feasibility of in-person group therapy is often limited by low attendance or high attrition [8, 13]. Common barriers to care in postpartum populations include childcare, transportation, and scheduling or time constraints [14–18]. This emphasizes the importance of alternative, including virtual, ways of delivering group-based treatments to ensure that these effective treatments are accessible to those who need them. At the onset of the COVID-19 pandemic, a rapid shift to the virtual delivery of group therapy occurred in many settings to ensure continuity of care as well as enhanced support during a very stressful and uncertain time. At Women’s College Hospital (WCH), an institutionally-driven approach was used whereby a videoconferencing service was securely integrated into the electronic health record in order to ensure the sustainable future delivery of group psychotherapy [19].

Little is known about the participant experience of group psychotherapy delivered in a virtual setting. A small (but growing) body of literature reports a wide spectrum of experiences with videoconferencing-based group therapy, suggesting that further research could increase our breadth of understanding about experiences overall, what underlies positive and negative experiences, and how these may vary among different populations [20–25]. Our institution intends on continuing to offer virtual groups as standard-of-care even as the COVID-19 pandemic recedes, to combat the other aforementioned barriers to care in this population. As such, understanding the participant experience within the virtual groups that we are delivering will help us best optimize their use to provide health care services at our institution. It will also help other institutions considering the use of videoconferencing-based group therapy services for the postpartum population. The objective of this study was to understand the experiences of those participating in synchronous, videoconferencing-based group therapy for postpartum depression and anxiety symptoms.

## Methods

### Setting

This study took place at WCH, a tertiary ambulatory care academic hospital located in Toronto, Ontario, Canada. WCH has a strong mandate related to the development and implementation of virtual solutions to support the equitable delivery of high-quality services within the context of Ontario’s publicly funded healthcare system. At the onset of the COVID-19 pandemic, WCH expedited the development and implementation of synchronous video-based group health interventions by integrating Zoom, a videoconferencing service, with EPIC and myChart, WCH’s electronic medical record system and patient-facing portal [19]. This enabled secure access to Zoom links for patients and providers, as well as complete clinical integration for scheduling, documentation, care planning, and follow-up. The immediate intention of this innovation was to provide continuity of care during the pandemic, and it was also developed and implemented in a manner that allowed for the continued and sustainable long-term delivery of videoconferencing group health services beyond the immediate needs of the pandemic.

### Study design and theoretical positioning

A pragmatic mixed-methods quality improvement evaluation on this videoconferencing group therapy intervention was conducted, including a quantitative survey [26] and this qualitative study. We used interpretive description and a social constructivist framework to develop an understanding of the experience of videoconferencing group participants and potentially use this information to inform future delivery of this program [27–29]. An interpretivist paradigm suggests that realities are subjective to the person experiencing it and in social constructivism, the meaning that someone ascribes to an experience is shaped by their interactions with others and the circumstances within which they live (i.e. their social world) [30]. In the context of this study, a shared experience (i.e. the videoconferencing therapy group) can be experienced in multiple ways by different individuals, depending on their previous lived experiences and how they perceive their interactions with others. Our positioning, therefore, allows us to gain insight into the experience of participating in the videoconferencing therapy group and its perceived impacts, based on the individual’s point of view and context. This approach has been used successfully in other studies related to perinatal mental health, including on digital health needs and the accessibility of mental health services for immigrant women [31, 32].

### Intervention

The Reproductive Life Stages Program (Department of Psychiatry) at WCH runs an 8-week psychotherapy group for women (cis and trans) and non-binary people assigned female sex at birth who are experiencing postpartum depression and anxiety symptomatology. Sessions are 60 min per week for eight weeks, with five to six participants and one highly-trained psychotherapist facilitator per videoconferencing group.

The group is interpersonal in nature, and its content and process loosely follows an interpersonal therapy (IPT) model, a psychotherapy modality that focuses on improving relationships with others to buffer the effect of major stressors on a person’s psychological health [33, 34], although it is not an IPT group. The therapy focuses on how relationships are being managed in any of 4 key focal areas: conflict in relationships, life changes, grief and loss, and difficulties in starting and maintain relationships [34]. The IPT model is particularly apt for the postpartum period, where people are sustaining major life changes, having to adapt to new expectations in relationships, and where social isolation is a major risk factor for the development and maintenance of illness [35]. In IPT, therapists are active and work with patients on specific problems, and to support options for action and change. During the course of the closed group (i.e. the same facilitator and group of patients for the duration of the 8 weeks) offered at WCH, the facilitator and patients engage in work including components of traditional IPT (e.g. education and discussion on losses and gains, the impact of transition and change, etc.), specific work on the role transition to parenthood, as well as guided mindfulness work. During the first session of each group, patients and their group facilitator collaboratively brainstorm topics for discussion which inform the specific theme of each week’s session. Conversation and socialization are important components of the group, and are emphasized due to the important role that social support plays in postpartum well-being. The group was offered in-person prior to March 2020 with free childcare offered onsite during in-person group sessions. Since May 2020, it has been offered virtually as standard-of-care, using the videoconferencing integration described above[26, 36]. After logging into the patient portal, patients complete an e-Check-in for the group and are automatically redirected to Zoom, where the facilitator admits them to the videoconferencing group.

### Participants, sampling, and recruitment

Upon referral to the Reproductive Life Stages program at WCH, all postpartum patients undergo a psychiatric assessment with a staff reproductive psychiatrist. At the end of that assessment, a treatment plan is formed, which may include referral to the postpartum depression and anxiety psychotherapy group. Group psychotherapy is the main psychotherapeutic modality offered for postpartum patients with depression and anxiety symptomatology in the program (approximately 50% of postpartum patients in our program are referred to group psychotherapy); individual therapy is only offered instead if the psychiatrist has concerns about participation in group therapy based on the clinical scenario (e.g. diagnosis, severity of illness). Referral to the postpartum depression and anxiety psychotherapy group does not require a specific diagnosis of a depressive or anxiety disorder, nor a specific symptom threshold on a depression or anxiety scale. The group can only be completed once per patient; post-group follow-up is provided by their psychiatrist as part of their treatment plan.

All patients who participated in the postpartum videoconferencing psychotherapy group between October 2021 and August 2022 (*n* = 134) were asked to complete a feedback survey. At the end of the survey, they were invited to optionally provide their contact information to participate in an in-depth interview to share more information on their experience in the group. All patients who provided this information were contacted in sequence (by phone and email) by NHS to participate in a qualitative interview. All group facilitators had, prior to the pandemic, provided in-person individual and group therapy services and had been working primarily virtually since the start of the pandemic. All facilitators were invited via email by NHS to complete an interview at a date and time of their convenience. All interviews were conducted between April-July 2022.

### Data collection

A semi-structured interview guide was developed for both patients and facilitators in order to ensure that relevant information related to the study objectives were captured (see Supplementary File 1). The interview guide was developed using the principles from the Mechanisms of Action in Group-Based Interventions (MAGI) Framework, which outlines the ways in which various components of group health interventions (e.g. intervention design, group dynamics and development, facilitation techniques, inter- and intra-personal change processes, and contextual influences) dynamically interact in order to influence outcomes [37, 38]. We also used the overall aims of the larger evaluation to guide the development of some research questions. Interviews were projected to be 45-60 min in length. The semi-structured approach allowed for the collection of data that was comparable between participants and yet flexible enough to explore other topics that arose during the interview, when the interviewee felt something was important to their experience. Interviews took place with a single interviewer (NHS) either on Zoom or by phone, depending on interviewee preference, were audio recorded, and then transcribed for analysis. All identifying information was removed from the transcripts which were also sent to each participant via email to ensure that they reflected their commentary accurately. The interviewer used reflexive memos during data collection to record important thoughts related to the interview in real-time, and to reflect shortly after the interview on the information gathered.

### Data analysis

Transcripts from patient participants were independently coded in NVivo 12 by two members of the study team (NHS and AS) using thematic analysis strategies typical of interpretivist qualitative research [39]. Specifically, a reflexive thematic analysis approach was used whereby after engaging in familiarization with the interview transcripts (i.e. data immersion, or reading and re-reading the transcripts), NHS and AS each independently engaged in deductive coding, based on the research aim of focusing on the experience of participating in virtual group therapy and the MAGI Framework [40]. Then, themes were constructed, revised and defined until it was felt that they best captured what was most meaningful from the data [40]. The two reviewers met periodically to ensure consistency in coding, compare analytic memos, and discuss themes, under the supervision of a senior researcher (JZ). Facilitator interviews were analysed as described above in tandem with the patient interviews, and were used to provide additional context and triangulate results.

### Ethical considerations

This initiative was formally reviewed by institutional authorities at WCH and was deemed not to require Research Ethics Board approval. It was reviewed and approved under the WCH Assessment Process for Quality Improvement Projects (APQIP #2021-0059-P). Potential participants were sent an email copy of the informed consent form prior to the interview, and verbal informed consent was obtained before interviews began. All interview participants were clearly informed that declining to participate in the qualitative interview (or stopping the interview early) would not affect their ability to receive care, nor will their circle of care at WCH be informed of their participation decision. They were informed that this research was part of NHS’ doctoral work, and had no other relationship with the interviewer. This study is reported using the Consolidated Criteria for Reporting Qualitative Studies guidelines [41].

### Reflexivity statement

Our study team was comprised of researchers (including trainees) and health care providers, all with varying levels of personal and professional experience delivering and/or receiving health care services virtually. Many of the health care providers specialize in mental health and have speciality training in perinatal mental health care, including those who facilitated the video groups and psychiatrists who are responsible for referring patients into group care. In addition, the study team was made up of women, many of whom are mothers. NHS, the primary author of this paper, designed this study under the supervision and mentorship of ES, SV, JZ and GM as part of her doctoral work.

## Results

### Participants

**Fig. 1:**
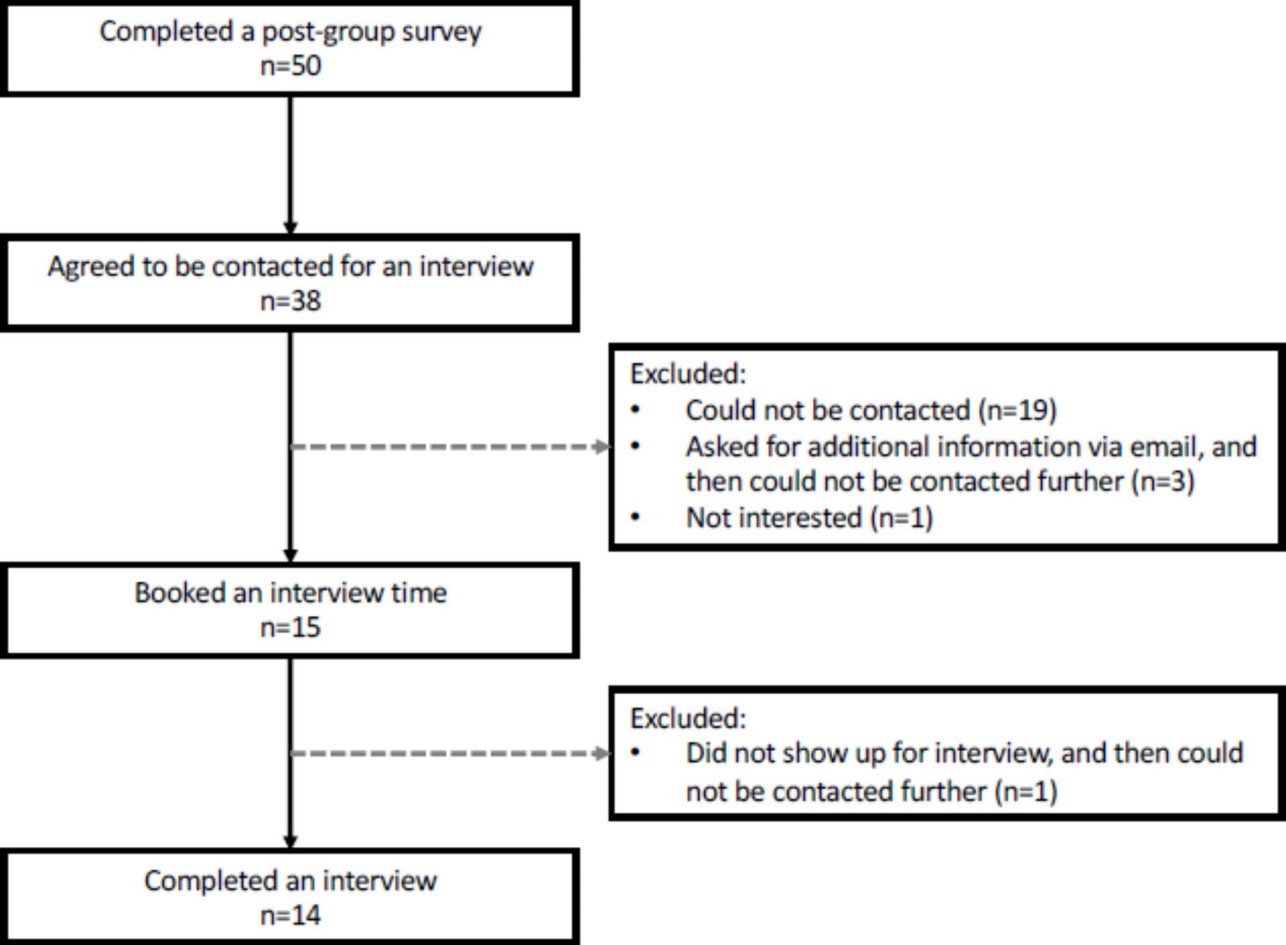
Diagram of participant recruitment

Of the 50 patients who completed the post-group survey, 38 provided their contact information and were contacted in sequence; 37% (*n* = 14/38) agreed to, and participated in an interview (Fig. 1).

All three facilitators completed an interview; facilitators had all been in practice for ≥ 7 years at the time of the interview, and were highly trained specialists in perinatal mental health (two were trained social worker therapists and one was a registered psychotherapist; supervision was not required). See Table 1 for patient participant demographics in comparison to those who completed the survey (additional facilitator demographics are not reported due to small sample size).

**Table 1:** Patient demographics. Demographics of patients who completed a qualitative interview (*N* = 14) compared to those who completed the survey (*N* = 50) as n (%), unless otherwise specified.

	**n (%)**	
	**Interview**	**Survey**
Age, mean (SD)	36.0 (3.8)	-
Woman	13 (92.9)	49 (98.0)
Heterosexual	12 (85.7)	39 (79.6)
Ethnicity (*select all that apply format*)		
Asian (East Asian, South Asian)	7 (50.0)	11 (22.0)
White	9 (64.3)	26 (52.0)
Other	3 (21.4)	13 (26.0)
Born in Canada	10 (71.4)	-
Married or Co-habitating	13 (92.8)	48 (96.0)
Number of children, mean (SD)	1.3 (4.7)	-
Age of youngest child in months, mean (SD)	10.6 (3.2)	6.3 (2.6)
Highest level of education		
≤ High school, College, technical school or undergraduate	7 (50)	-
Graduate degree	7 (50)	-
Household income		
≤ $119,999	6 (42.9)	15 (30.0)
≥ $120,000	8 (57.1)	27 (54.0)

**Fig. 2:**
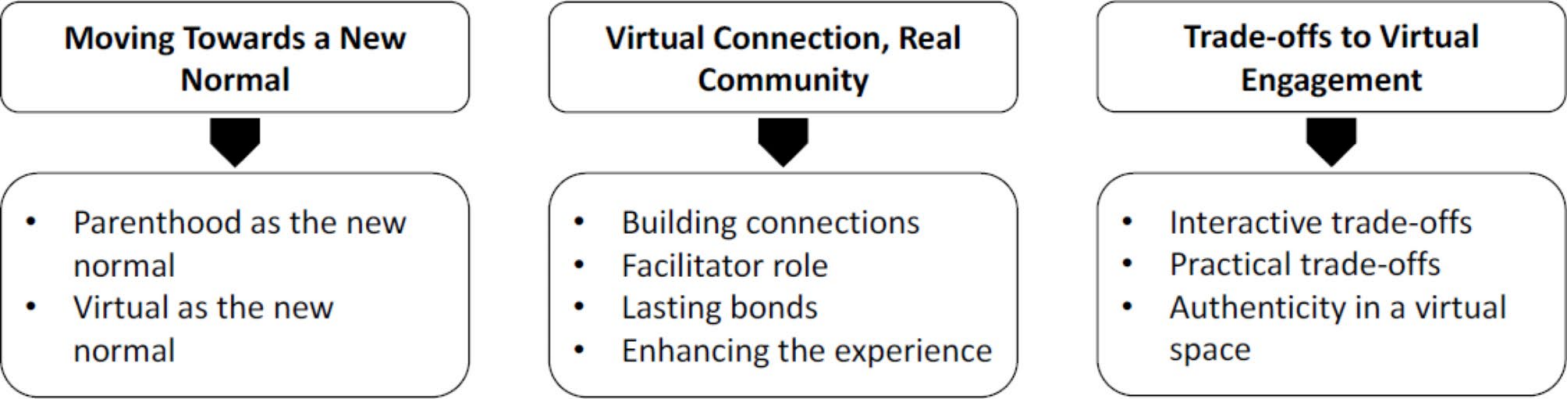
Overview of themes

Three main themes were constructed, each with additional sub-themes (see Fig. 2).

### Theme 1: Moving towards a new normal

There were two main elements of change that led participants to this video group: the pandemic and the subsequent move to delivering the group virtually, and the shift to parenthood. Participants in the interviews described their experience with the videoconferencing group as one that was normal or normalizing, in two distinct ways. First, participants found that their engagement with the group helped them normalize their feelings around the transition to parenthood. Second, participants acknowledged that virtual health care was now a normal part of the way in which health care is received.

#### Parenthood as the new normal

Through the conversations that took place during the virtual group, participants came to understand that many of their difficulties adjusting to parenthood were shared by others, thus reducing their anxieties and concerns; they were able to learn from others and understand that they were part of a shared experience transitioning to parenthood. One participant described this experience:

*“The feeling was just like we’re just going to let it all hang out in this room and we’re going to be able to celebrate our wins and also complain about things that suck [laughs]. And also, it’s fine, this is just normal. So really just maybe go from anxious to just like [sighs] OK, I’m just a mom and I’m just a new mom and we’re just going to do life, me and my son and my husband, we’re just going to do things that work for us. So it just really brought me down to a more grounded place.”* (Participant 08)

Another participant described the group’s impact on her ability to identify with many of the challenges faced by others during this transition to parenthood, and seeing her experience as a normal one:

*“Hearing other people’s stories always made me feel better because it really did contextualize what I’m experiencing this larger experience of motherhood that is very – is varied and it’s – and a breadth of experiences all of which are normal and OK and – so that was helpful to me, hearing other people’s challenges I was – many of those challenges resonated with me.”* (Participant 10)

#### Virtual as the new normal

Participants acknowledged that the use of virtual tools like Zoom to access healthcare in general, and participate in the virtual group specifically, were now a part of everyday life. For most, this stemmed from the fact that by the time they participated in the group, they had already been using virtual tools for quite some time for work and social purposes due to the pandemic. As one participant said:

*“You know, [Zoom is] something we kind of had to all learn how to use very quickly, you know, being in an office setting once the pandemic began. I felt comfortable.”* (Participant 05)

This is not to say that participants were always in favour of using virtual tools to access care. Some still preferred in-person interactions and most appreciated the option of using virtual tools when circumstances warranted its use. One group participant noted the dichotomy between wanting to engage in in-person healthcare and preferring the convenience of virtual:

*“I would have preferred to see them [other healthcare providers] in person. However in addition to the pandemic situation it would have been difficult for me to get to, like I don’t have a car, and I don’t always have child care… so this just made it a lot more accessible for me… I was given the option recently of going to see my GP or having a virtual appointment and I chose the virtual one.”* (Participant 03)

### Theme 2: Virtual connection, real community

#### Building connections

Participants reflected on the fact that although their groups were conducted entirely virtually, they felt like they were able to develop meaningful and real connections with other group members. Most participants noted that, prior to starting the virtual group, they had high expectations and desires to build connections and relationships with other virtual group participants. For some, this was reflective of their experiences being isolated during the pandemic (both prior to and during their pregnancy), and for others this reflected a desire to create the “village” that is colloquially discussed in the context of parenthood. As one participant recalled their anticipation:

*“I was more so excited – excited is a little bit of a strong word but I was more so looking forward to chatting with some other moms that were probably going through something similar to me, just to feel some sort of – some more connection and some more support from those individuals to feel less alone or less, like, crazy.”* (Participant 14)

Developing connections with other participants virtually was not something that came naturally to most participants. For many, it took time to feel comfortable enough to share intimate details and feel connected to others online. One participant discussed the challenges with creating connections virtually:

*“Zoom is great for a lot of things, but for that kind of unspoken like physical connection it maybe is lacking at times. And maybe people didn’t feel as comfortable sharing, you know, in front of a computer until the conversation picked up. And usually that would occur halfway through the meeting and then we’d almost be out of time; but there’d still be like lots more to say.”* (Participant 01)

Another participant reflected on the fact that, despite being connected virtually, the similarities between group members helped foster their bonds:

*“I feel like even though it was virtual there was still a connection there, because we had common ground.”* (Participant 04)

This sentiment was echoed by another:

*“Building the virtual relationship, it was different but it still felt like organic and it felt pretty easy because we were all kind of there for I think the same thing and the same support.”* (Participant 14)

For others, building virtual connections was hard:

*“It didn’t feel like a different interaction, I would close down Zoom and maybe open up another tab, it just felt like another tab on the internet rather than a group of people.”* (Participant 11)

#### Facilitator role

Regardless of individual feelings about creating relationships with others in the virtual group, the important and impressive role played by the group therapist in facilitating the creation of a sense of community (e.g. by highlighting commonalities among different experiences) was discussed by several participants:

*“So someone might say something about what they were going through that week, and I might say something that I thought was, you know, a different experience, and she would find a way to connect them. And I think what it did is it created a sense of community, and it certainly kind of made me feel like what I was saying had value for what other people were experiencing.”* (Participant 01)

Another participant reflected on the highly skilled nature of the therapist, and her ability to conduct the group in a way that is flexible to adjust to participant needs:

*“You don’t have the impression that she’s running through a set of questions that she has jotted down on a piece of paper. She’s following the group and when a topic had been exhausted or everyone had checked in, she would come up with something that usually worked well to get us going again. And that’s a skill, that’s not automatic.”* (Participant 07)

Facilitators themselves reflected on the nuances of leading a group virtually and the role they play in setting the stage for patients to interact with one another:

*“We open the group and then we close the group at the end. And… we try to be… pretty flexible, try to be very casual, you know, in terms of allowing discussion in however it’s going to kind of organically come about. You know, not super-structured in terms of, like, this is the topic, or like, we have to speak and then the next person speaks and then the next. You know, there’s going to be a little bit of overlap and that’s OK… But there’s a formality that comes with it.”* (Participant F02)

Another facilitator acknowledged that while they do play a part in helping participants navigate these new relationships virtually, their role only goes so far:

*“I love when a group really clicks. That I can’t – you know I do my very best, right, to encourage those relationships no matter what but there are some groups of people that come together, you can’t really coordinate that chemistry, right, it’s an interpersonal chemistry.”* (Participant F01)

#### Lasting bonds

Participants felt that although their relationships with other group members were created in a virtual space, meaningful and lasting bonds were created that extended beyond the reach of the group. Some strong bonds were between individuals. One participant, regarding another group member reaching out to her to keep in touch at the end of the group, said:

*“I found her really funny and honest. Funny in this, like, unintentional way. She was just so frank all the time… like, she was just so honest about her experience and I just – I really liked it. So I was happy that she reached out. And I really liked – I think we’ve ended up having more in common than I initially thought we did. So that’s been really good. And yeah, it was something I’d hoped for. I hoped that I would make like a friend, a friend in some respects.”* (Participant 05)

Other bonds were created among the group as a whole. Another participant said:

*“And it actually created some lasting friendships, we decided as a group to create our own little forum in which we meet at the same time on Thursdays like we used to. Yeah and sometimes those things happen in group and then it fall aways but they seem to be sticking so that’s been nice.”* (Participant 08)

Facilitators recognized the participant desire to keep their group connections beyond the virtual space:

*“So, although they don’t have that little [casual interaction] before and that little [casual interaction] bit after, people are really expressing early on, I think, wanting to connect after, because that’s often something that happens; people form their own groups and stay connected afterwards, and that’s also been something that people have really taken up quite a bit.”* (Participant F02)

#### Enhancing the experience

Although the virtual connection was meaningful, participants had suggestions for how to enhance and strengthen the community-building experience. Many offered practical suggestions, including the desire to have a longer group time (e.g. the length of the weekly session, or increased number of weeks), which they felt would provide additional time to “warm up” to the virtual experience in order to feel more comfortable sharing their personal experiences which each other; many enjoyed the video group and wanted a longer duration simply to extend the experience. These suggestions are not entirely new or unique to the group in its virtual format, as one facilitator acknowledged:

*“We’ve always had that feedback, for the last 12 years, no matter what the length of… [the group] was, everyone’s always said they wish it went on for longer. They almost always say that as the group is ending, they don’t say that when we first enroll them. When we enroll them they’re like, “Well that’s a long time, I don’t know if I can commit to that.” And then once they realize that they like it and it’s working for them they want it to go on.”* (Participant F01)

Other practical suggestions were to connect with other participants offline during the course of the group:

*“Some kind of method of communication with other participants during the course of the group therapy. We were offered the option to connect after it was over, but you know, sometimes something would occur to me and I’d want to discuss it with another mom, and I’d have to wait a whole week. And I’d often forget.”* (Participant 01)

Some participants highlighted the need for more diverse representation within the group. For example, few LGBTQ + , single parent, and BIPOC families were represented. Some participants from more marginalized groups felt unable to fully share the spectrum of their experience in group because they felt like they could not fully relate to or be understood by those who did not share their lived experience and suggested having groups tailored to those who shared certain characteristics. As one facilitator noted:

*“Should we be creating groups that are more sort of strategically curated in terms of particular demographics like, you know, LGBTQ group,… BIPOC-identified group, you know, first time parent, second time parents[?] Like that’s something that we try to balance. We do try to have at least two second time parents in a group because otherwise they feel like their experience is very different.”* (Participant F01)

### Theme 3: Trade-offs to virtual engagement

Pervasive through the participant experience was the notion that there were positive and negative trade-offs when it came to the lived experience of the virtual group, versus the potential perceived benefits of an in-person one.

#### Interactive trade-offs

One noted trade-off was the lack of informal interactions before and after the virtual session that may have occurred during an in-person session. Many participants felt like they missed out on the potential opportunity to casually bond as they arrived and exited the clinic:

*“If there were other women who live around the area we might realize hey are you walking in the same direction and like, you know, build some organic conversations in like that. I would have loved it… I think it would have been great; but if the group were in the wintertime that’s another story.”* (Participant 10)

However, other participants brought up the benefit of a virtual group in eliminating the need for small talk, which may have contributed to the group being able to dive in to the deeper issues that brought each participant to the group:

*“It allows you to cut through the social niceties. And in not knowing the people but in kind of coming together for the same reason, I think you’re able to be a bit more blunt about sharing your experiences.”* (Participant 01)

#### Practical trade-offs

Participants recognized that there were many practical benefits of participating in the virtual group with an infant. For example, not having to manage travel and transportation, particularly in the context of distance to the hospital and weather, were often cited as reasons for their consistent attendance and participation:

*“If had to choose one or the other I probably would choose virtual just because it works around my baby’s schedule and I don’t have to go out downtown or try to ride the bus, it just makes it more convenient. But there is something special and magical about in-person things, whatever it is. I just don’t think for me that it could trump the convenience of online, at this time anyway.”* (Participant 08)

On the other hand, some noted that it would have been beneficial for them and their mental health to have a reason to leave the house with their infant, as a virtual group still left them feeling physically isolated:

*“With the virtual experience, there is still a bit of, it’s still very artificial, it’s still, it’s not as effective at getting to that, yeah, breaking that sense of isolation, when you’re still kind of just in your house on your computer.”* (Participant 11)

An ongoing trade-off to the ease of in-home access to the group surrounded the matter of childcare. For some, it was a distraction to have their infant around while they participated in the group, whereas others felt that the flexibility of attending to baby’s needs during the group (e.g. putting baby down for a nap, feeding, etc.) was a major bonus. Some participants enlisted the help of a family member to look after the infant during the group time. One participant said:

*“I just found it completely stressful… I found that having [my child] there didn’t help me have the moment that I needed. You know it wasn’t then about me – it wasn’t time I was taking for myself… I couldn’t fully connect to the group… there were a couple of times I had to have her there and then at some point I’d SOS my husband and be like, you need to take her. And it was always better when she wasn’t there. So I had to kind of coordinate some kind of, for lack of a better way of putting it, childcare for her. Even if it was for half the session I found that that’s what I needed. And sometimes it’s stressful. Like, sometimes it took a lot to get there. But never regretted it.”* (Participant 05)

This was validated by one of the group facilitators who noted the difficulty that comes with holding the attention of participants in a virtual environment:

*“…Sometimes they’re multitasking. And that reduces the ability for me to kind of build a connection, because they might not be on screen as often, or they might kind of be focusing on like, wrangling their crawling baby. And so sometimes I find that there is this like disconnection that happens when the attention shifts from being sort of in the group, to sort of splitting the attention between being in the group, potentially participating, and then taking care of their child.”* (Participant F03)

#### Authenticity in a virtual space

The nature of a virtual group provided some unique added value in terms of helping participants gain insight into what the everyday reality was like for others; this cannot be replicated within in-person groups. Participants noted that had the group been in person, they would have come well-dressed (e.g. nicer clothes, wearing makeup, hair done), but because the group was accessed from home, they showed up in a more physically authentic way. By the same token, they appreciated catching glimpses into the lives of others in the group; seeing what life was like for others in the context of parenthood made them feel better about themselves.

*“It was clear that… nobody else had it any more together than I did, so in that sense. You know, nobody else had a particularly tidy space, nothing looked like an Instagram profile, nothing – you know, there wasn’t a single baby who didn’t cry at some point. There wasn’t a single baby who wasn’t adorable at some point. There wasn’t a single time when somebody didn’t have to turn off the camera for a sec and go do a thing.”* (Participant 07)

## Discussion

Patients in our study described the unique experience of participating in synchronous, videoconferencing-based group psychotherapy for postpartum depression and anxiety. The change agents of motherhood and of the pandemic and growth of virtual care, were normalized for participants, who balanced competing needs in order to participate and experience a virtual connection with other mothers in their group (Fig. 2). Their experience demonstrates that while virtual care is understood to be part of the general healthcare experience now, there are both benefits and challenges to the experience. Overall, there was a sense that these new mothers enjoyed and benefitted from the group. A virtual experience may have partially blunted the ability to create meaningful in-group connections for some, but with a skilled facilitator a positive group environment and sense of cohesion could still be achieved. Interestingly, no one mentioned preferring virtual therapy to reduce the risk of disease (e.g. COVID) transmission; these interviews were conducted in the spring and summer of 2022 among individuals who participated between autumn 2021 and spring 2022. Most benefits of the group related to the common barriers to accessing treatment that have been noted in the literature, which demonstrates that videoconferencing-based group therapy has a place in addressing those barriers for postpartum women, beyond its usefulness in a pandemic context.

The findings of this study complement existing research on the functioning of (in-person) groups for healthcare purposes. The MAGI Framework, as discussed above, suggests that intervention design, group dynamics and development, facilitation techniques, inter- and intra-personal change processes, and contextual influences all dynamically interact with each other to influence outcomes in health related groups; this framework was developed in the context of in-person group interventions, and our study provides data to suggest that it may also be applicable to groups that take place in a virtual environment [37, 38]. In our study, positive group dynamics and a strong sense of connection can be achieved in a virtual environment. The literature on group climate in a virtual context is mixed with some studies (both quantitative and qualitative) demonstrating good levels of video-based group cohesion and others a lower one, although these studies do acknowledge that lower cohesion is offset when considering overall outcomes due to factors such as higher attendance [42–45]. Studies highlight the important role of facilitators in tailoring their skills to the online environment, and in using specific tools that are unique to videoconferencing platforms (e.g. breakout rooms, virtual whiteboards, etc.) in order to help foster connection between members; participants in our study emphasized the excellence of their group facilitators which may, in part, help to explain our finding on positive group climate. More research is needed to understand the nuances of developing group cohesion in a videoconferencing group and factors that may help promote this.

Participants in the present study reflected that their interpersonal connections to others in the group helped reduce their worries related to motherhood, and helped them feel more comfortable with their changing identities and new roles. This finding is similar to those found in other studies on in-person postpartum groups, further validating the appropriateness of video group therapy in this population [46]. The transition to motherhood is a complex one, involving intra-personal transformation in multiple domains, including the physical, psychological, social, and relational [47, 48]. This is a significant process and life stage which can be lonely, particularly for those managing symptoms of postpartum depression or anxiety in relation to this shift [35]. The literature highlights that peer support and validation from healthcare professionals (both of which are elements of the group in this evaluation) can ameliorate a sense of loneliness, as can support from others in one’s social network (which can be strengthened using skills developed as a result of the group) [35]. Our data also demonstrated the unique value-add of virtual groups in allowing participants to feel more comfortable in attending authentically, and gain insight into the lived realities of others that would not have occurred in person.

Virtual mental health therapy has been touted as a way of addressing barriers to accessing care in this and other populations, and our study demonstrates that tensions around barriers to care still exist even when care is offered virtually. For example, our study notes that while participants appreciated not having to travel into the hospital for their therapy group, participants noted that they still felt physically isolated when participating in a videoconferencing group. Similarly, the voices of our participants suggest that when accessing group therapy virtually, childcare-related matters still had to be negotiated in order to engage in the group. For example, some participants enjoyed being able to have their infants with them while others had to either ask their partner or family to look after the infant, or manage their own lack of focused attention on the group content. These contextual factors that are unique to the postpartum context, therefore, continue to play a role in accessing virtual group therapy. Our study echoes the findings of a recent scoping review on the opportunities and challenges of group video-based health interventions (e.g. lack of non-verbal communication), and highlights other nuances that may be specific to the postpartum population (e.g. normalizing what home life looks like in early motherhood, lasting connections to counter the isolation of motherhood, and the challenges of childcare) [49].

### Strengths and Limitations

While there is qualitative literature on the experience of perinatal mental health video group facilitators, and of patients who transitioned to videoconferencing groups during the pandemic, our study is unique in its examination of the experience of participants in a synchronous, video-based group therapy for women with postpartum depression and anxiety symptoms [24, 25]. Strengths of the study include its unique population of participants in videoconferencing group therapy for postpartum depression and anxiety symptoms within the context of a hospital that promotes and values the sustainable use of virtual tools to deliver high quality care; therefore study results will be used to help improve ongoing virtual health care programming. Those who conducted the thematic analysis (NHS and AS under the supervision of JZ) were not involved in the design or delivery of the group, reducing the risk of bias in our interpretation of data. Limitations of the present study include that a convenience sample of women who were active participants in the group were used; we were unable to speak to those who dropped out of the videoconferencing group to understand their experiences and the reason for their drop-out. In addition, our sample was relatively homogenous with limited representation from equity-deserving groups. Finally, this study has a small sample size of patients, although interviews were conducted until data saturation was achieved.

## Conclusions

Participants in our study highlight the important ways in which they felt value from videoconferencing-based group psychotherapy for postpartum depression and anxiety symptoms. Participants were able to foster meaningful connections with other group members and achieve a better grasp of their own mental health and transition to motherhood. Results of our study will help inform the continued development and improvement of video therapy groups as a way of delivering mental health services for the postpartum population. Future research should look at differences that may lead some to derive maximum benefit from in-person versus video group therapy in order to inform choice in mental health treatment and health service delivery.

## Supplementary Information

Supplementary Material 1. Interview_Guides. Shows the semi-structured interview guides used for both patient and provider interviews.

## Data Availability

No datasets were generated or analysed during the current study.

## Abbreviations

WCH: Women’s College Hospital
IPT: Interpersonal Therapy
MAGI: Mechanisms of Action in Group-Based Interventions Framework
