# Supplementary material for: Virtual connection and real community: the qualitative experience of participating in a videoconferencing-based psychotherapy group for postpartum depression and anxiety
Source: BMC Health Serv Res. 2024 Nov 1;24:1329. doi: 10.1186/s12913-024-11753-y (PMC11529178; doi:10.1186/s12913-024-11753-y)
Supplement: Supplementary file 1 — Supplementary Material 1. Interview_Guides. Shows the semi-structured interview guides used for both patient and provider interviews. [file 12913_2024_11753_MOESM1_ESM.docx]

Table of Contents

[Interview Guide for Patients 1](#_Toc71194533)

[Interview Guide for Providers 4](#_Toc71194534)

## Interview Guide for Patients

**Set-Up and Introduction**

Thank you for taking the time to talk with me today. My name is Neesha and I am a PhD Student at the University of Toronto. I am working on a study to understand the outcomes and experiences of those who have participated in the Virtual Interpersonal Therapy Group for Postpartum Women. You may recall that you have already completed two questionnaires for this study. The findings from this research will be used to help understand how virtual groups can be used to deliver mental health care to women in the perinatal period like yourself, and to people who may not otherwise be able to access in-person therapy groups.

This interview will take approximately 30 minutes. I will be asking questions about your experience with this group, and your opinion on how we might use this treatment, delivered through video chat, in the future. There are no right or wrong answers, or desirable or undesirable answers. I would like you to feel comfortable saying what you really think and how you really feel. If you do not want to answer any question, you can say "pass".

Before we start the interview, do you have any questions?

*[Pause for questions and provide answers as appropriate]*

As you know, an audio recorder will be used for our interview to ensure that I have accurately captured what you have said. It can be stopped at any time; just let me know if you'd like it to be stopped. Does this sound okay?

[*Participant confirms recording is OK, NHS will turn recorder on.*]

For the purpose of the recording, I would like to state that today is [*date*] and this is my [*time*] interview with [*participant number*].

**Background Information**

1. Can you tell me about your baby?
   1. How old are they?
   2. Can you tell me more about who lives at home with you?
2. You recently completed the Interpersonal Therapy Group for Postpartum Women with the Reproductive Life Stages Program at Women’s College Hospital. Can you tell me about what you were experiencing that led you to enroll in this group?
3. You accessed this group through an online platform.
   1. How comfortable are you with technology and digital tools (like computers, the internet, and smartphones) in general?
   2. Have you used any types of technology or digital tools (like apps, websites, smart watches, telemedicine) for your health before?
      1. What did you use these for? What did you like or dislike about your experiences with them?

**Program-Specific Inquiry**

1. Now that I know a little bit about you and your experiences, I would like to know in general, what was your experience like participating in this group?
2. With regards to participating in a virtual group therapy program:
   1. What were your concerns? Were these realized?
   2. What were you looking forward to? Did this happen?
3. What was it like to access group therapy online?
   1. What was the process for logging in like? What did you use to log in (i.e. the type of device)? Where did you participate from (e.g., home, etc.)?
   2. How did this compare to what you thought it would be?
4. What was virtual group participation like when it came to:
   1. The facilitator: What did you think when you first met her in the group? What did you think about the way in which she facilitated the group?
   2. The group members: What was it like to develop a relationship with other group members online? What were your interactions like?
   3. How was your experience of group therapy different than other, individual supports you have?
5. In the past groups like this were offered in person. How do you think this experience would have been similar or different in person?
   1. One of the reasons why this group is offered virtually is because of the pandemic. Has the pandemic changed your views on using virtual care to get health care? In what way?
6. How well did you feel this group addressed your overall needs?
   1. How could the virtual group be improved?

**Sensitizing Concepts**

If the participant introduces any of the following concepts, the interviewer will ask open-ended clarifying and exploratory questions pertaining to that topic:

1. Experiences of group therapy: maintaining privacy and confidentiality vs participating in a shared experience; Yalom’s therapeutic factors (altruism, cohesion, universality, interpersonal learning input and output, guidance, catharsis, identification, family re-enactment, self-understanding, hope, existential factors); developing connection virtually.
2. Experiences of virtual care: technological equity; diversity and marginalization; building rapport and connection virtually; comfort, convenience and accessibility.
3. Experiences related to PPD/PPA: isolation; role transition.
4. Experiences related to the pandemic: fear (of getting sick, going to the hospital); isolation; financial concerns; changes to normal supports; screen fatigue.

If any of the above are identified, the following questions can be used to follow up:

1. Can you tell me more about that (person, event)?
2. Can you give me a specific example?
3. Can you explain your answer?
4. In what way?
5. How did you understand that?
6. What does that mean to you?

## Interview Guide for Providers

**Set-Up and Introduction**

Thank you for taking the time to talk with me today. My name is Neesha and I am a PhD Student at the University of Toronto. I am working on a study to understand the outcomes and experiences of those who have participated in the Virtual Interpersonal Therapy Group for Postpartum Women. You may recall that you have already completed two questionnaires for this study. The findings from this research will be used to help understand how virtual groups can be used to deliver mental health care to women in the perinatal period, and to people who may not otherwise be able to access in-person therapy groups.

This interview will take approximately 30 minutes. I will be asking questions about your experience with facilitating this group virtually, and your opinion on how we might how we might use this treatment, delivered through video chat, in the future. There are no right or wrong answers, or desirable or undesirable answers. I would like you to feel comfortable saying what you really think and how you really feel. If you do not want to answer any question, you can say "pass".

Before we start the interview, do you have any questions?

*[Pause for questions and provide answers as appropriate]*

As you know, an audio recorder will be used for our interview to ensure that I have accurately captured what you have said. It can be stopped at any time; just let me know if you'd like it to be stopped. Does this sound okay?

[*Participant confirms recording is OK, NHS will turn recorder on.*]

For the purpose of the recording, I would like to state that today is [*date*] and this is my [*time*] interview with [*participant number*].

**Background Information**

1. You recently completed facilitating an Interpersonal Therapy Group for Postpartum Women. I’d like to know about your experience with facilitating these groups virtually.
   1. How comfortable are you with technology and digital tools (like computers, the internet, and smartphones) in general?
   2. What was it like to facilitate this group virtually, in comparison to your previous experience facilitating this group in person?

**Structured Inquiry**

1. What was it like to deliver group therapy online?
   1. What was it like to prepare for the group beforehand, compared to how you would prepare for an in-person group?
   2. How did this compare to what you thought it would be?
2. What was your experience of facilitating the virtual group like when it came to:
   1. Developing a relationship with each of the group members?
   2. Fostering relationships between group members?
3. How do you feel the members of the group experienced the virtual care group?
   1. How do you feel virtual group therapy delivers care in comparison to in-person group therapy?
   2. What surprised you about it?
4. What were your concerns about facilitating a virtual group therapy program? What were you looking forward to with a virtual group therapy program?
5. How well did you feel this group was able to address patient needs?
   1. How could the virtual group be improved?
6. One of the reasons why this group is offered virtually is because of the pandemic. Has the pandemic changed your views on using virtual care to get health care? In what way?

**Sensitizing Concepts**

If the participant introduces any of the following concepts, the interviewer will ask open-ended clarifying and exploratory questions pertaining to that topic:

1. Experiences of group therapy: maintaining privacy and confidentiality vs participating in a shared experience; Yalom’s therapeutic factors (altruism, cohesion, universality, interpersonal learning input and output, guidance, catharsis, identification, family re-enactment, self-understanding, hope, existential factors); developing connection virtually.
2. Experiences of virtual care: technological equity; diversity and marginalization; building rapport and connection virtually; comfort, convenience and accessibility.
3. Experiences related to the pandemic: fear (of getting sick, going to the hospital); isolation; financial concerns; changes to normal supports; screen fatigue.

If any of the above are identified, the following questions can be used to follow up:

1. Can you tell me more about that (person, event)?
2. Can you give me a specific example?
3. Can you explain your answer?
4. In what way?
5. How did you understand that?
6. What does that mean to you?
